# Anti-Inflammatory Effects of Flavonoids: Genistein, Kaempferol, Quercetin, and Daidzein Inhibit STAT-1 and NF-κB Activations, Whereas Flavone, Isorhamnetin, Naringenin, and Pelargonidin Inhibit only NF-κB Activation along with Their Inhibitory Effect on iNOS Expression and NO Production in Activated Macrophages

**DOI:** 10.1155/2007/45673

**Published:** 2007-08-20

**Authors:** Mari Hämäläinen, Riina Nieminen, Pia Vuorela, Marina Heinonen, Eeva Moilanen

**Affiliations:** ^1^The Immunopharmacology Research Group, University of Tampere, Medical School and Tampere University Hospital, Research Unit, Tampere 33014, Finland; ^2^Department of Biochemistry and Pharmacy, Åbo Akademi University, Turku 20520, Finland; ^3^Department of Applied Chemistry and Microbiology, University of Helsinki, Helsinki 00014, Finland

## Abstract

In inflammation, bacterial products and proinflammatory cytokines induce the formation of large amounts of nitric oxide (NO) by inducible nitric oxide synthase (iNOS), and compounds that inhibit NO production have anti-inflammatory effects. In the present study, we systematically investigated the effects of 36 naturally occurring flavonoids and related compounds on NO production in macrophages exposed to an inflammatory stimulus (lipopolysaccharide, LPS), and evaluated the mechanisms of action of the effective compounds. Flavone, the isoflavones daidzein and genistein, the flavonols isorhamnetin, kaempferol and quercetin, the flavanone naringenin, and the anthocyanin pelargonidin inhibited iNOS protein and mRNA expression and also NO production in a dose-dependent manner. All eight active compounds inhibited the activation of nuclear factor-κB (NF-κB), which is a significant transcription factor for iNOS. Genistein, kaempferol, quercetin, and daidzein also inhibited the activation of the signal transducer and activator of transcription 1 (STAT-1), another important transcription factor for iNOS. The present study characterises the effects and mechanisms of naturally occurring phenolic compounds on iNOS expression and NO production in activated macrophages. The results partially explain the pharmacological efficacy of flavonoids as anti-inflammatory compounds.

## 1. INTRODUCTION

Nitric oxide (NO) is produced from L-arginine by three nitric oxide synthase (NOS) enzymes; endothelial NOS (eNOS), neuronal NOS (nNOS), and inducible NOS (iNOS). Low physiological levels of NO are produced by constitutively expressed eNOS and nNOS, whereas iNOS is responsible for prolonged production of larger amounts of NO. iNOS is induced by bacterial products and inflammatory cytokines in macrophages and some other cells [1–3]. NO production is increased in inflammation and has proinflammatory and regulatory effects [4, 5]. In addition, peroxynitrite formation in a reaction of NO and superoxide may lead to increased cytotoxicity. The experimental data support the idea that compounds inhibiting expression or activity of iNOS are potential anti-inflammatory agents [6–9].

Flavonoids are naturally occurring polyphenolic compounds containing two benzene rings linked together with a heterocyclic pyran or pyrone ring. Flavonoids are normal constituents of the human diet and are known for a variety of biological activities. Some of these act as enzyme inhibitors and antioxidants, and have been reported to have anti-inflammatory properties. However, the molecular mechanisms explaining how flavonoids suppress the inflammatory response are not known in detail [10, 11]. There are studies showing that certain flavonoids down-regulate NO production in response to inflammatory stimuli [12–14], but no more precise mechanisms of action are known.

In the present study, we investigated the effects of 36 naturally occurring compounds representing different groups of flavonoids and related compounds on iNOS expression and NO production in activated macrophages systematically, and evaluated the mechanisms of action of the effective compounds.

## 2. MATERIALS AND METHODS

### 2.1. Materials

Luteolin, luteolin-7-glucoside, vitexin, daidzein, genistein, genistin, rhamnetin, isorhamnetin, kaempferol, myricetin, taxifolin, naringin, ferulic acid, pelargonidin, procyanidin B1, and procyanidin B2 were obtained from Extrasynthese (Lyon, France). Acacetin, cyanidin, flavone, morin, and quercitrin were obtained from Carl Roth GmbH (Karlsruhe, Germany). Quercetin, rutin, and benzoic acid were obtained from Merck (Darmstadt, Germany). Naringenin, +catechin, −epicatechin, ellagic acid, gallic acid, and syringic acid were obtained from Sigma (St. Louis, MO, USA). Apigenin, chlorogenic acid, dodecyl gallate, methyl gallate, octyl gallate, and sinapic acid were obtained from Fluka (Buchs SG, Switzerland).

Dulbecco's modified eagle medium and its supplements were obtained from Gibco BRL (Paisley, UK). All other reagents were obtained from Sigma (St. Louis, MO, USA) unless otherwise stated.

### 2.2. Cell culture

Murine J774 macrophages were obtained from American Type Culture Collection (Rockville, MD, U.S.A). Cells were cultured at 37°C (in 5% carbon dioxide) in Dulbecco's modified eagle's medium (DMEM) with glutamax-I containing 10% heat-inactivated foetal bovine serum, penicillin (100 units/ml), streptomycin (100 *μ*g/ml), and amphotericin B (250 ng/ml). Cells were harvested with trypsin-EDTA. Cells were seeded in 96-well plates for XTT-test, in 24-well plates for nitrite measurements, in 6-well plates for iNOS Western blot and RNA extraction, and in 10-cm dishes for p65 and STAT-1α Western blot. Confluent cells were exposed to fresh culture medium containing the compounds of interest and cultured for the times indicated.

### 2.3. XTT-test

Cell viability was tested using cell proliferation kit II that measures the cells' ability to metabolize XTT to formazan by mitochondrial dehydrogenase activity, a function that only occurs in viable cells (Roche Diagnostics GmbH, Mannhein, Germany). Cells were incubated with the tested compounds and LPS for 20 hours before addition of sodium 3′-[1-(phenylaminocarbonyl)-3,4-tetrazolium]-bis (4-methoxy-6-nitro) benzene sulphonic acid hydrate (XTT) (final concentration 0.3 mg/ml) and N-methyl dibenzopyrazine methyl sulphate (8.2 *μ*M). Cells were incubated for another 4 hours and the amount of formazan accumulating in growth medium was assessed spectrophotometrically. Triton-X treated cells were used as a positive control. Conditions were considered nontoxic if the cells' ability to metabolize XTT to formazan was more than 80% of that of cells exposed to LPS only.

### 2.4. Nitrite determinations

Measurement of nitrite accumulation into the culture medium was used to determine NO production. At the indicated time points, the culture medium was collected and nitrite was measured by the Griess reaction [15]. A NOS inhibitor L-NIO (1 mM) and a highly selective iNOS inhibitor 1400 W (1 mM) were added at the beginning of the incubation to cells that were stimulated with LPS (100 ng/ml) to ensure that the measured nitrite was due to NO produced by the iNOS pathway in the cell culture.

### 2.5. Preparation of cell lysates for iNOS Western blot

After the desired time of incubation cell lysates were prepared as described earlier [16]. The Coomassie blue method was used to measure the protein content of the samples [17].

### 2.6. Preparation of nuclear extracts for p65 and STAT-1α Western blot

Cells were seeded on 10-cm dishes and were grown to confluence. Cells were incubated with the compounds of interest for 30 minutes (p65) or for 6 hours (STAT-1α). After incubation, samples were prepared as described earlier [18]. The Coomassie blue method was used to measure the protein content of the samples [17].

### 2.7. Western blot analysis of iNOS, p65, and STAT-1α proteins

Protein samples (20 *μ*g) were separated by SDS-PAGE on 8% polyacrylamide gel and transferred to nitrocellulose membrane. Bound antibody (rabbit polyclonal antibodies for iNOS, STAT-1α (Santa Cruz Biotechnology, Santa Cruz, CA, USA), or for p65 subunit of NF-κB (Cell Signaling Danvers, MA, USA)) was detected using goat anti-rabbit polyclonal antibody (Santa Cruz Biotechnology, Santa Cruz, CA, USA), and visualised by SuperSignal chemiluminescent substrate (Pierce, Cheshire, UK) and FluorChem 8800 imaging system (Alpha Innotech Corporation, San Leandro, CA). The quantitation of the chemiluminescent signal was carried out using FluorChem software version 3.1.

### 2.8. RNA extraction and real-time RT-PCR of iNOS and GAPDH mRNAs

J774 cells stimulated with the compounds of interest were trypsinised after the desired time of incubation. Cell homogenization, RNA extraction, reverse transcription, and quantitative PCR were performed as described earlier [16]. The primer and probe sequences and concentrations were optimized according to the manufacturer's instructions in TaqMan Universal PCR Master Mix Protocol part number 4304449 revision C and were as follows: 5′-CCTGGTACGGGCATTGCT-3′, 5′-GCTCATGCGGCCTCCTT-3′, (forward and reverse mouse iNOS primers, resp., both 300 nM), 5′-CAGCAGCGGCTCCATGACTCCC-3′ (mouse iNOS probe containing 6-FAM as 5′-reporter dye and TAMRA as 3′-quencher, 150 nM), 5′-GCATGGCCTTCCGTGTTC-3′, 5′-GATGTCATCATACTTGGCAGGTTT-3′ (forward and reverse mouse glyceraldehyde-3-phosphate dehydrogenase (GAPDH) primers, resp., both 300 nM), 5′-TCGTGGATCTGACGTGCCGCC-3′ (mouse GAPDH probe containing 6-FAM as 5′-reporter dye and TAMRA as 3′-quencher, 150 nM). Results of iNOS mRNA levels were normalized against GAPDH mRNA in each sample.

### 2.9. Statistics

Results are expressed as mean ± standard error of mean (SEM). The statistical significance of the detected differences was calculated by analysis of variance followed by Dunnett multiple comparison's test. Differences were considered significant when P<.05.

## 3. RESULTS

**Figure 1:**
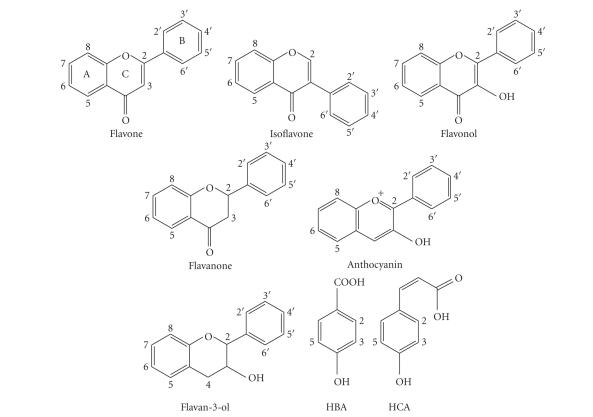
Basic chemical structures of the phenolic compounds used in the present study.

The tested compounds (n=36) represented eight groups of flavonoids and related compounds: flavones, isoflavones, flavonols, flavanones, flavan-3-ols, anthocyanins, hydroxybenzoic acid (HBA) group, and hydroxycinnamic acid (HCA) group. The tested compounds are listed and their structures are shown in Figure 1 and Table 1 . Possible cytotoxic effects were tested by XTT-test. Compounds that were toxic at 100 *μ*M (see Table 2) were excluded from further studies.

### 3.1. Effects of flavonoids on LPS-induced NO production in J774 cells

Untreated J774 macrophages did not produce detectable amounts of NO during 24-hour incubation, but LPS (100 ng/ml) enhanced NO production significantly. In the first experiments, flavonoids were used at 10 *μ*M and 100 *μ*M concentrations. The compounds inhibiting NO production by more than 50% at 100 *μ*M concentration compared to LPS-treated control were flavone, daidzein, genistein, isorhamnetin, kaempferol, quercetin, naringenin, and pelargonidin (see Table 2). NOS inhibitor L-NIO (1 mM) and a selective iNOS inhibitor 1400 W (1 mM) were used as control compounds, and they inhibited LPS-induced NO production by more than 90%.

**Figure 2:**
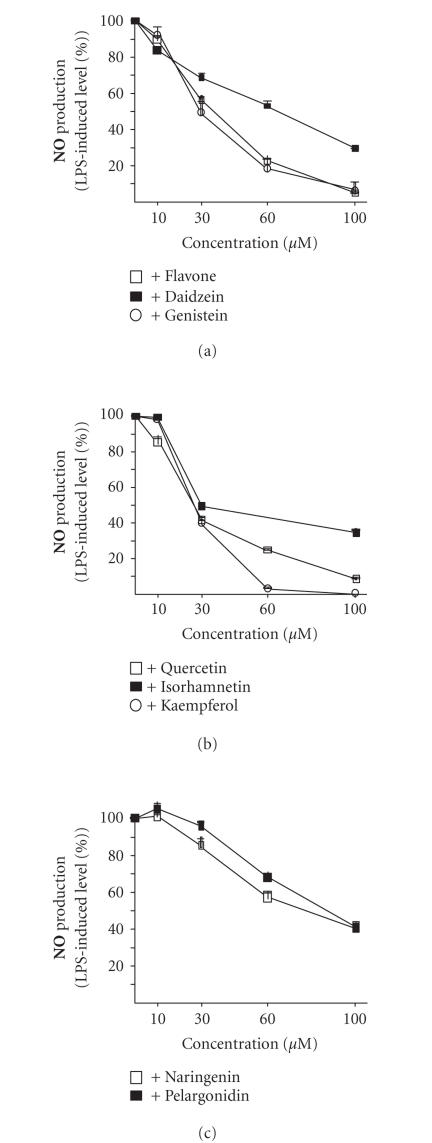
(a) Effects of increasing concentrations of flavone, daidzein, and genistein on LPS (100 ng/ml)-induced NO production in J774 cells during a 24-hour incubation time. (b) Effects of increasing concentrations of quercetin, isorhamnetin, and kaempferol on LPS (100 ng/ml)-induced NO production in J774 cells during a 24-hour incubation time. (c) Effects of increasing concentrations of naringenin and pelargonidin on LPS (100 ng/ml)-induced NO production in J774 cells during a 24-hour incubation time. NO production was determined by measuring nitrite accumulation in the culture medium by Griess reaction. Mean + SEM, n=6.

If the compound inhibited NO production by more than 50% at 100 *μ*M concentration, a dose-response effect was studied. All eight active compounds (flavone, daidzein, genistein, isorhamnetin, kaempferol, quercetin, naringenin, and pelargonidin) inhibited NO production in a dose-dependent manner in the following order: quercetin (IC_50_ ∼ 25 *μ*M) ∼ kaempferol (IC_50_ ∼ 25 *μ*M) > genistein (IC_50_ ∼ 30 *μ*M) ∼ isorhamnetin (IC_50_ ∼ 30 *μ*M) > flavone (IC_50_ ∼ 40 *μ*M) > daidzein (IC_50_ ∼ 70 *μ*M) > naringenin (IC_50_ ∼ 80 *μ*M) > pelargonidin (IC_50_ ∼ 90 *μ*M) (see Figure 2).

### 3.2. Effects of flavonoids on LPS-induced iNOS protein expression

**Figure 3:**
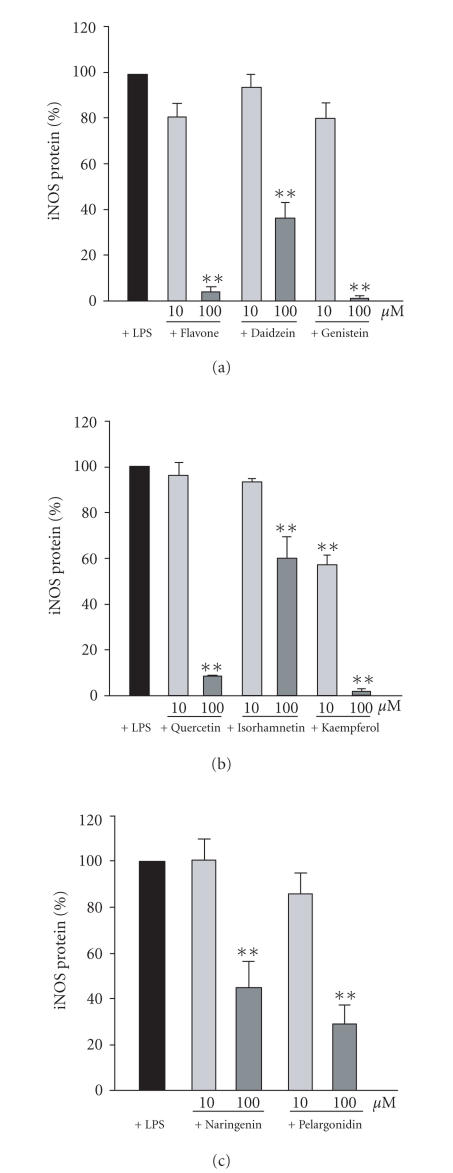
(a) Effects of flavone, daidzein, and genistein on LPS (100 ng/ml)-induced iNOS protein expression in J774 cells during a 24-hour incubation time. (b) Effects of quercetin, isorhamnetin, and kaempferol on LPS (100 ng/ml)-induced iNOS protein expression in J774 cells during a 24-hour incubation time. (c) Effects of naringenin and pelargonidin on LPS (100 ng/ml)-induced iNOS protein expression in J774 cells during a 24-hour incubation time. iNOS protein expression was detected by Western blot. Mean + SEM, n=3, **P<.01.

The effects of those eight flavonoids inhibiting NO production by more than 50% at 100 *μ*M concentrations were tested on iNOS protein expression by Western blot analysis. Unstimulated cells did not express detectable amounts of iNOS protein and LPS enhanced iNOS protein expression considerably. All eight active compounds (flavone, daidzein, genistein, isorhamnetin, kaempferol, quercetin, naringenin, and pelargonidin) inhibited LPS-induced iNOS protein expression (see Figure 3).

### 3.3. Effects of flavonoids on LPS-induced iNOS mRNA levels

**Figure 4:**
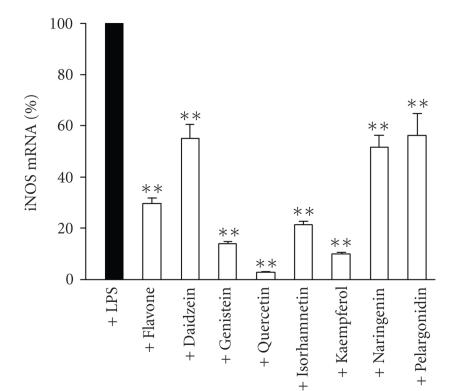
Effects of flavonoids on iNOS mRNA expression. Cells were treated with LPS (100 ng/ml) or with LPS and the tested compound (100 *μ*M) and RNA was extracted after 6 hours. iNOS and GAPDH mRNA were measured by real-time RT-PCR and iNOS mRNA levels were normalised against GAPDH. Mean + SEM, n=3, **P<.01.

iNOS mRNA was measured by quantitative real-time RT-PCR. Cells were incubated with LPS (100 ng/ml) or with LPS and the tested flavonoid (flavone, daidzein, genistein, isorhamnetin, kaempferol, quercetin, naringenin, or pelargonidin (100 *μ*M)) for 6 hours. This incubation time was chosen according to the time curve of iNOS mRNA, where the maximal iNOS mRNA levels were between 6 and 8 hours after addition of LPS. Untreated cells expressed very low levels of iNOS mRNA and LPS enhanced iNOS mRNA expression considerably. All eight tested flavonoids significantly lowered iNOS mRNA levels when measured after 6-hour incubation in the following order of potency: quercetin > kaempferol > genistein > isorhamnetin > flavone > naringenin > daidzein ∼ pelargonidin (see Figure 4).

### 3.4. Effects of flavonoids on LPS-induced activation of transcription factors NF-κB and STAT-1

NF-κB and STAT-1 are important transcription factors for iNOS [19, 20]. Therefore we measured the effects of the eight effective flavonoids on NF-κB and STAT-1 activations by measuring the nuclear translocation of the factors by Western blot.

**Figure 5:**
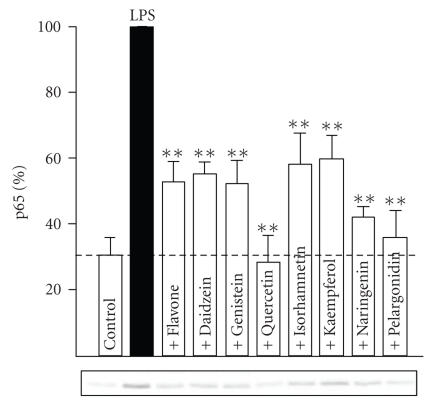
Effects of flavonoids on LPS-induced NF-κB activation determined as nuclear translocation of NF-κB. J774 cells were incubated for 30 minutes with LPS (100 ng/ml) or with LPS and the flavonoid (100 *μ*M), and nuclear proteins were extracted. Western blot was used to detect the p65 subunits of NF-κB in the nuclear extracts. p65 levels in LPS-treated cells were set at 100% and the other values were related to that. The dotted line represents the nuclear p65 levels in untreated control cells. Mean + SEM, n=4−6, **P<.01 as compared to LPS-induced level.

In unstimulated cells, low basal activity of NF-κB was detected and was significantly enhanced after LPS challenge. The maximal activation was found 30 minutes after LPS addition, and that incubation time was used in the subsequent studies. Quercetin, naringenin and pelargonidin inhibited the LPS-induced activation of NF-κB by more than 80%. Flavone, genistein, isorhamnetin, kaempferol, and daidzein had a moderate (57%–72% inhibition) inhibitory effect (see Figure 5).

**Figure 6:**
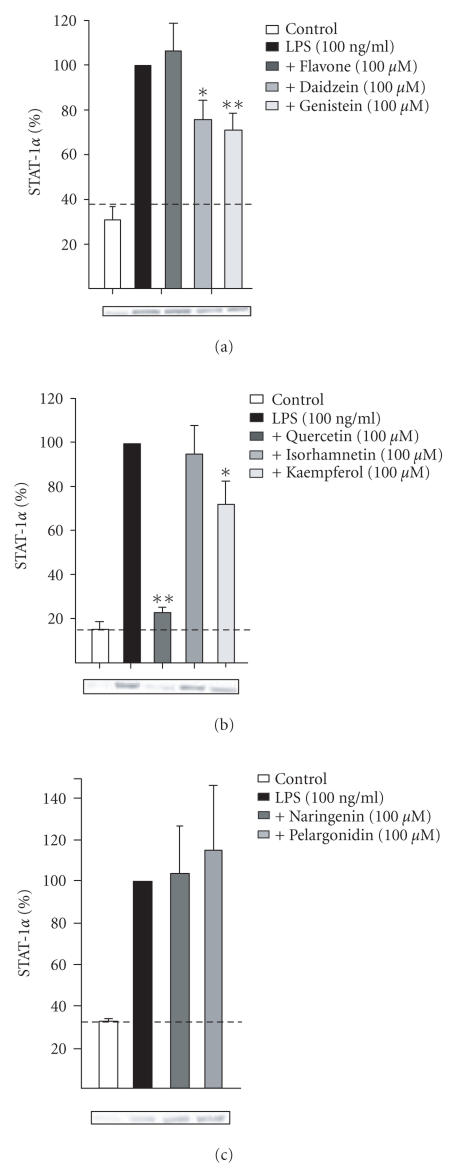
(a)–(c) Effects of flavonoids on LPS-induced STAT-1 activation determined as nuclear translocation of STAT-1α. J774 cells were incubated for 6 hours with LPS (100 ng/ml) or with LPS and the flavonoid (100 *μ*M), and nuclear proteins were extracted. Western blot was used to detect STAT-1α in the nuclear extracts. STAT-1α levels in LPS-treated cells were set at 100% and the other values were related to that. The dotted line represents the nuclear STAT-1α levels in untreated control cells. Mean + SEM, n=3, ** P<.01, * P<.05 as compared to LPS-induced level.

Nuclear STAT-1 levels were significantly enhanced after LPS challenge. The maximal effect was found 6 hours after LPS addition, and that time point was chosen for subsequent studies. The LPS-induced activity of STAT-1 was nearly totally (91% inhibition) inhibited by quercetin. Daidzein, genistein, and kaempferol had a moderate (32%–41% inhibition) inhibitory effect whereas flavone, isorhamnetin, naringenin, and pelargonidin showed no inhibitory effect on the activation of STAT-1 (see Figure 6).

## 4. DISCUSSION

Flavonoids are nonessential dietary factors, and humans consume about 1-2 g of flavonoids daily. Flavonoids are abundantly present in fruits, vegetables, seeds, nuts, tea, and red wine, and the flavonoid mostly consumed is quercetin. Flavonoids are believed to act as health-promoting substances, and some of them have antioxidant and anti-inflammatory properties [10, 11]. Anti-inflammatory effects have also been found in vivo. For instance, genistein was reported to inhibit LPS-induced septic response in rat [21] and quercetin suppressed experimentally induced arthritis in rat [22].

In the present study, we investigated the effects of flavonoids and related compounds belonging to eight classes (flavones, isoflavones, flavonols, flavanones, flavan-3-ols, anthocyanins, HBA, and HCA) on iNOS expression and NO production in activated macrophages. Eight effective compounds were found. Four compounds (genistein, kaempferol, quercetin, and daidzein) inhibited LPS-induced STAT-1 and NF-κB activations, and iNOS expression. In addition, four compounds (flavone, isorhamnetin, naringenin, and pelargonidin) inhibited NF-κB activation and iNOS expression but had no effect on STAT-1.

Isoflavones daidzein and genistein inhibited LPS-induced iNOS expression and NO production in a dose-dependent manner, whereas genistin was less effective. Daidzein and genistein also inhibited activations of STAT-1 and NF-κB, which are important transcription factors for iNOS [19, 20]. To our knowledge, their effects on STAT-1 activation have not been reported previously, whereas suppression of DNA-binding of NF-κB by genistein has been reported [23]. Our results confirm earlier observations on the inhibitory effects of daidzein and genistein on iNOS expression and NO production [13, 24–27], and provide a mechanism for the effect through suppression of STAT-1 and NF-κB activations.

In the flavonol group, isorhamnetin, kaempferol, and quercetin inhibited NO production and iNOS protein and mRNA expression, quercetin and kaempferol being the most potent of the phenolic compounds tested. Isorhamnetin, kaempferol, and quercetin all inhibited NF-κB activation, and quercetin and kaempferol also had an effect on STAT-1 activation. This is the first study to show that isorhamnetin reduces iNOS expression, and that the effect may well be mediated by inhibition of NF-κB activation. Kaempferol has previously been shown to inhibit iNOS expression and NO production [13, 28]. Here we confirm those findings and show that kaempferol inhibits STAT-1 and NF-κB activations, which are implicated in their effects on iNOS expression. Chen et al. [29] have reported that quercetin inhibits IFN*γ*-induced STAT-1 activation in mouse BV-2 microglia. In the present study we found that quercetin also suppressed LPS-induced activation of STAT-1 in macrophages supporting the idea that its effects on STAT-1 are stimulus and cell-type independent. Quercetin inhibited LPS-induced STAT-1 activation along with its inhibitory effect on iNOS expression and NF-κB activation. Inhibition of STAT-1 activation by quercetin is likely involved in the mechanisms by which it inhibits iNOS expression because JAK inhibitors AG-490 and WHI-P154 have been shown to inhibit iNOS expression along with their suppressive actions on STAT-1 activation [18, 30].

Concurring with earlier results, we found flavanone naringenin to inhibit LPS-induced NO production while its glycosylated counterpart naringin had no effect [31]. Our results suggest that the inhibitory effect of naringenin is likely to be at transcriptional level through inhibition of the activation of NF-κB. Pelargonidin has been reported to inhibit NO production in macrophages [26]. Here we extend the data by showing that pelargonidin suppresses NO production by reducing iNOS expression through inhibiting the activation of transcription factor NF-κB.

Regarding the structural requirements of flavonoids for the inhibition of NO production, three main features could be found:

(a) a C-2,3 double bond is a common feature in the six most effective compounds,

(b) a bulky group (e.g., glycoside, rhamnoside, rutinoside, or neohesperidoside) as a substituent lowered or abolished the compound's inhibitory effect (e.g., quercetin was highly effective whereas its rhamnoside-substituted derivative quercitrin was ineffective),

(c) 7 and 4′ OH-groups were found in all effective compounds but this alone did not differentiate active from ineffective compounds.

Related structure-activity relationships regarding PGE_2_ inhibition have been reported in rat peritoneal macrophages [32].

Earlier studies have shown that some flavonoids inhibit NO production in response to inflammatory stimuli [13, 26, 28, 33, 34]. The present study extends the previous knowledge by systematically comparing the effects of a large series of compounds in standardized experimental conditions. Moreover, we investigated the effects of the eight effective compounds not only on NO production but also on iNOS mRNA and protein expression, and on the activation of inflammatory transcription factors NF-κB and STAT-1. Some anti-inflammatory flavonoids have been shown to inhibit activation of NF-κB and the effect has been linked to their antioxidant properties [29, 35]. Here we found that all the effective compounds (flavone, daidzein, genistein, isorhamnetin, kaempferol, quercetin, naringenin, and pelargonidin) inhibited LPS-induced NF-κB activation. In addition, genistein, kaempferol, quercetin, and daidzein also inhibited STAT-1 activation. To our knowledge, the inhibitory effects of genistein, kaempferol, and daidzein on STAT-1 activation have not been reported previously, whereas quercetin was found to inhibit IFN*γ*-induced activation of STAT-1 in mouse BV-2 microglia [29]. The mechanisms by which genistein, kaempferol, quercetin, and daidzein inhibit STAT-1 activation are not known, but may be associated with inhibition of phosphorylation of STAT-1 or its up-stream kinase JAK2 [36]. Interestingly, the three most potent inhibitors of iNOS expression and NO production, that is, genistein, kaempferol, and quercetin, inhibited both NF-κB and STAT-1 activations, whereas those flavonoids inhibiting only NF-κB had a smaller effect on iNOS expression. Because NF-κB and STAT-1 are involved in the activation of several inflammatory genes, flavonoids that inhibit activation of NF-κB and/or STAT-1 are likely to down-regulate production of an array of inflammatory mediators in addition to iNOS. Therefore the present results offer an additional molecular mechanism for the anti-inflammatory action of flavonoids.

In conclusion, we compared the effects of 36 naturally occurring flavonoids and related polyphenolic compounds on LPS-induced NO production and iNOS expression in activated macrophages. The flavonoid classes containing the most effective compounds were isoflavones and flavonols. We identified eight compounds as being able to inhibit LPS-induced NO production and iNOS expression. Four compounds (genistein, kaempferol, quercetin, and daidzein) inhibited activation of both of the important transcription factors for iNOS, that is, STAT-1 and NF-κB, whereas four compounds (flavone, isorhamnetin, naringenin, and pelargonidin) inhibited only NF-κB. The results partly explain the anti-inflammatory effects of flavonoids.

## Figures and Tables

**Table 1:** Chemical structures of the phenolic compounds used in the present study.

Class	Derivatives	Substituents							
		1 *	3	5	7	3′	4′	5′	
Flavones	Acacetin		H	OH	OH	H	OCH_3_	H	
	Apigenin		H	OH	OH	H	OH	H	
	Flavone		H	H	H	H	H	H	
	Luteolin		H	OH	OH	OH	OH	H	
	Lut-7-glucoside		H	OH	OGlc	OH	OH	H	
	Vitexin		H	OH	OH	H	OH	H	8 Glc

Isoflavones	Daidzein		H	H	OH	H	OH	H	
	Genistein		H	OH	OH	H	OH	H	
	Genistin		H	OH	OGlc	H	OH	H	

Flavonols	Isorhamnetin		OH	OH	OH	OCH_3_	OH	H	
	Kaempferol		OH	OH	OH	H	OH	H	
	Morin		OH	OH	OH	H	OH	H	2′ OH
	Myricetin		OH	OH	OH	OH	OH	OH	
	Quercetin		OH	OH	OH	OH	OH	H	
	Quercitrin		ORha	OH	OH	OH	OH	H	
	Rhamnetin		OH	OH	OCH_3_	OH	OH	H	
	Rutin		ORu	OH	OH	OH	OH	H	

Flavanones	Naringenin		H	OH	OH	H	OH	H	
	Naringin		H	OH	ONeo	H	OH	H	
	Taxifolin		OH	OH	OH	OH	OH	H	

Flavan-3-ols	+Catechin		OH 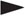	OH	OH	OH	OH	H	
	−Epicatechin		OH 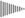	OH	OH	OH	OH	H	
	Procyanidin B1		Dimer of epicatechin and catechin linked via their carbons 4 and 8, respectively.						
	Procyanidin B2		Dimer of two epicatechin molecules linked via carbons 4 and 8.						

Anthocyanins	Cyanidin		OH	OH	OH	OH	OH	H	
	Pelargonidin		OH	OH	OH	H	OH	H	

HBA	Benzoic acid		H	H					4 H
	Dodecyl gallate	COO(CH_2_)_11_CH_3_	OH	OH				
	Ellagic acid	see below							
	Gallic acid		OH	OH					
	Methyl gallate	COOCH_3_	OH	OH				
	Octyl gallate	COOCH_2_(CH_2_)_6_CH_3_	OH	OH				
	Syringic acid		OCH_3_	OCH_3_			

HCA	Chlorogenic acid	see below							
	Ferulic acid		OCH_3_					
	Sinapic acid		OCH_3_	OCH_3_					

*if other than in basic structure

Glc = glycoside, Rha = rhamnoside, Ru = rutinoside, Neo = neohesperidoside

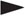
 OH group is in front of plane of paper, 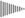 OH group is behind plane of paper

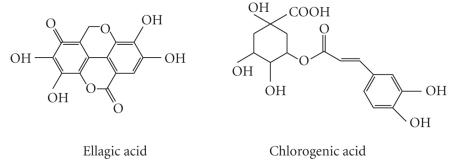

**Table 2:** Effects of phenolic compounds on cell viability and on LPS-induced (100 ng/ml) NO production in J774 macrophages. No detectable NO production was found in untreated cells. Mean ± standard error of mean (SEM).

Class	Toxicity^(a)^	NO production (inhibition%)	
Derivatives	[100 *μ*M]	[10 *μ*M]	[100 *μ*M]

***LPS 100 ng/ml***		0	0
**Flavones**			
Acacetin	+		
Apigenin	+		
Flavone	−	20.4±1.7	92.8±0.6
Luteolin	+		
Luteolin-7-glucoside	−	3.1±1.7	38.6±1.1
Vitexin	−	−1.9±0.8	6.2±0.7

**Isoflavones**			
Daidzein	−	11.3±6.3	70.3±3.1
Genistein	−	11.6±2.4	97.4±0.2
Genistin	−	9.7±2.1	13.3±2.0

**Flavonols**			
Isorhamnetin	−	3.5±0.9	65.1±1.6
Kaempferol	−	8.4±2.2	99.6±0.2
Morin	−	4.8±1.3	40.8±2.4
Myricetin	−	−0.6±2.6	32.3±2.8
Quercetin	−	−1.5±5.1	89.7±0.3
Quercitrin	−	5.1±0.8	19.1±0.8
Rhamnetin	+		
Rutin	−	−2.2±2.0	8.7±1.0

**Flavanones**			
Naringenin	−	14.7±1.3	59.6±3.3
Naringin	−	1.9±2.5	3.9±2.1
Taxifolin	−	3.1±1.8	23.9±1.8

**Flavan-3-ols**			
+ Catechin	+		
− Epicatechin	−	−0.6±1.8	−0.1±1.0
Procyanidin B1	−	−8.4±1.4	−2.2±1.9
Procyanidin B2	−	−0.6±2.5	0.8±1.7

**Anthocyanins**			
Cyanidin	−	−4.9±3.8	5.3±1.8
Pelargonidin	−	4.5±1.1	59±0.8

**HBA** ^(b)^			
Benzoic acid	−	0.7±2.0	−4.0±1.5
Dodecyl gallate	+		
Ellagic acid	−	−15.1±3.0	38.2±1.9
Gallic acid	+		
Methyl gallate	−	11.2±0.9	30.5±1.0
Octyl gallate	+		
Syringic acid	−	6.7±1.5	5.1±1.6

**HCA** ^(c)^			
Chlorogenic acid	−	−4.8±3.5	−0.9±2.0
Ferulic acid	−	6.6±1.1	9.0±1.5
Sinapic acid	−	8.9±1.9	5.2±1.3

	n=6	n=6	n=6

^(a)^Cytotoxicity was tested by XTT-test and compounds that showed cytotoxicity at 100 *μ*M concentrations were excluded from further study. ^(b)^HBA = hydroxybenzoic acid, ^(c)^HCA = hydroxycinnamic acid, n = number of replicates.
